# High-Performance Solid-State Thermionic Energy Conversion Based on 2D van der Waals Heterostructures: A First-Principles Study

**DOI:** 10.1038/s41598-018-27430-0

**Published:** 2018-06-18

**Authors:** Xiaoming Wang, Mona Zebarjadi, Keivan Esfarjani

**Affiliations:** 1 Department of Physics and Astronomy, The University of Toledo, Toledo, Ohio 43606 United States; 2 Wright Center for Photovoltaic Innovation and Commercialization, The University of Toledo, Toledo, Ohio 43606 United States; 3 Department of Electrical and Computer Engineering, University of Virginia, Charlottesville, Virginia 22904 United States; 4 Department of Materials Science, University of Virginia, Charlottesville, Virginia 22904 United States; 5 Department of Mechanical and Aerospace Engineering, and University of Virginia, Charlottesville, Virginia 22904 United States; 6 Department of Physics, University of Virginia, Charlottesville, Virginia 22904 United States

## Abstract

Two-dimensional (2D) van der Waals heterostructures (vdWHs) have shown multiple functionalities with great potential in electronics and photovoltaics. Here, we show their potential for solid-state thermionic energy conversion and demonstrate a designing strategy towards high-performance devices. We propose two promising thermionic devices, namely, the p-type Pt-G-WSe_2_-G-Pt and n-type Sc-WSe_2_-MoSe_2_-WSe_2_-Sc. We characterize the thermionic energy conversion performance of the latter using first-principles *GW* calculations combined with real space Green’s function (GF) formalism. The optimal barrier height and high thermal resistance lead to an excellent performance. The proposed device is found to have a room temperature equivalent figure of merit of 1.2 which increases to 3 above 600 K. A high performance with cooling efficiency over 30% of the Carnot efficiency above 450 K is achieved. Our designing and characterization method can be used to pursue other potential thermionic devices based on vdWHs.

## Introduction

Solid-state thermionic energy conversion was proposed by Mahan *et al*.^1,2^ and Shakouri *et al*.^3^ about 20 years ago. Working as refrigerators or power generators, they are competitive to^4,5^, or could be even better than^6^ thermoelectric modules. We note that the key to distinguish the thermionic and thermoelectric devices is the carrier transport regime. While the former is dominated by ballistic transport, the latter operate in the diffusive regime. In ballistic transport, the transport channel is shorter than the electron mean free path. Therefore, the dimension along the transport direction should be at nanoscale (electron mean free path) to meet the requirement of the ballistic transport for the thermionic devices. Solid-state thermionic devices have several advantages over their vacuum counterpart including lower operating temperatures, absence of space charge effect, easy access to cathode and anode for the purpose of cooling and heating and finally higher reliability and easier fabrication due to the absence of the vacuum. The main drawback of the solid-state thermionic modules is the heat leakage through the lattice vibrations of the semiconductor barrier layers, which is much stronger compared to the radiation heat leak in vacuum thermionic modules^7^. Hence, semiconductors with large thermal resistance and large thermal contact resistance are desirable to achieve high device performance, e.g., the cooling efficiency of about 30% can be achieved with the thermal resistance parameter *T*_R_ of 200–300 K^1^ which is equivalent to a thermal conductance *C*_*th*_ of 4–9 MW m^−2^ K^−1^. Such small thermal conductance values at nanoscale (<100 nm) can be obtained with the emergence of van der Waals heterostructures (vdWHs) composed of vertically stacked two-dimensional (2D) materials^8,9^. For example, we calculated *C*_*th*_ to be 4–6 MW m^−2^ K^−1^ for graphene-phosphorene-graphene vdWH sandwiched by gold electrodes^10^. In another work^11^ a *C*_*th*_ as low as 0.5 MW m^−2^ K^−1^ was experimentally estimated for a graphene-WSe_2_-graphene vdWH. A molecular dynamics study^12^ evaluated a slightly higher *C*_*th*_ of 17 MW m^−2^ K^−1^ for both graphene-WSe_2_-graphene and graphene-MoSe_2_-graphene vdWHs. The unprecedented low thermal conductance has renewed interests in solid-state thermionic energy conversion of vdWHs in recent years^10,12–14^.

Most of theoretical work on thermionic energy conversion uses the Richardson’s law for the thermionic current $J=A{T}^{2}{e}^{-{E}_{b}/{k}_{B}T}$, where $A=e{m}^{\ast }{k}_{B}^{2}\bar{\tau }/(2{\pi }^{2}{\hslash }^{3})$ is the Richardson constant, *e* is the electron charge, m^*^ is the electron effective mass, $\bar{\tau }$ is the averaged electron transmission denoting the fraction of electrons transmitted from the metal to the semiconductor, *k*_*B*_ is the Boltzmann constant, $\hslash$ is the reduced Planck constant, *T* is the absolute temperature, and *E*_*b*_ is the thermionic barrier height which is usually taken as a parameter to optimize the thermionic energy conversion efficiency^1,2,12^. A small *E*_*b*_ of several *k*_*B*_*T* is found to be optimal for a single barrier thermionic energy converter^1^.

In the case of 2D vdWHs, one can benefit from the large database of 2D semiconductors with different electron affinity^15^. In addition, due to quantum confinement effects, 2D materials have layer-dependent band alignment^16^. Therefore, one can tune *E*_*b*_ by changing the number of layers. However, evaluating *E*_*b*_ of the vdWH-based thermionic device is nontrivial due to the Fermi level pinning effect at the metal-2D material interface^17^. The interfacial property also affects $\bar{\tau }$ and *C*_*th*_, both of which are interface dependent and cannot be estimated easily. We note that all the above mentioned difficulties can be remedied by the parameter-free density functional theory (DFT) based first-principles calculations. First-principles study of solid-state thermionic energy conversion, though rarely reported^10^, holds great potential in the field due to the strong predictive power of DFT.

A great challenge for DFT to calculate the thermionic transport in vdWHs is to accurately evaluate *E*_*b*_, which is directly related to the bandgap of the semiconductor layer. DFT usually underestimates the semiconductor bandgap due to the self-interaction error. In addition, the bandgap of 2D materials shows large renormalization due to the substrate or dielectric screening^18–20^, a dynamic polarization effect not captured by DFT^21^. This would overestimate the bandgap. The two effects seem to cancel each other to some extent but not completely. The accurate calculation of *E*_*b*_ is of great importance since the thermionic current changes exponentially with *E*_*b*_ and therefore the device performance is quite sensitive to *E*_*b*_. To this end, we use the *GW* approximation^22^ to calculate the quasiparticle band structures and the band alignment of the vdWHs.

**Figure 1:**
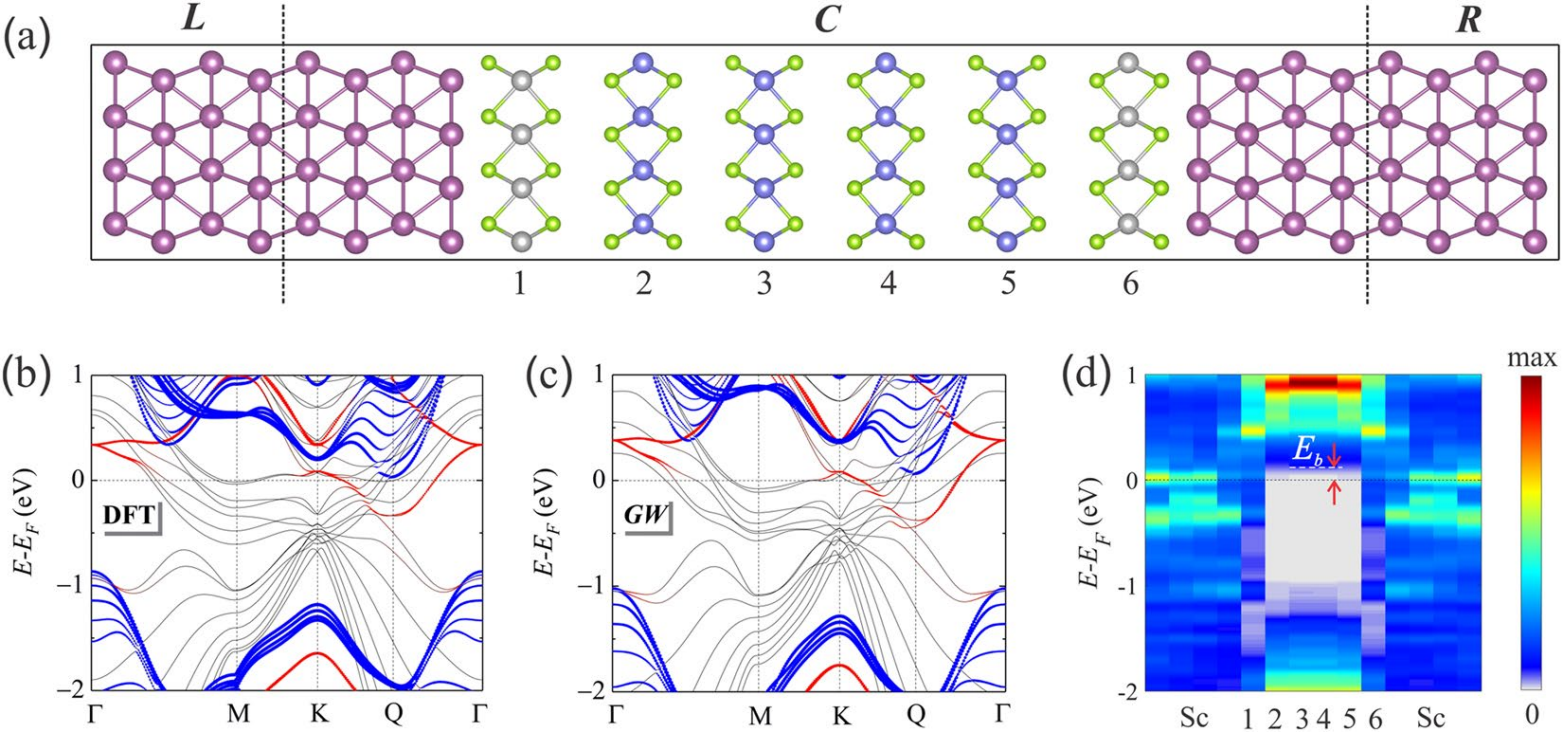
(**a**) Ball-stick model of the proposed device configuration. *L* and *R* are two semi-infinite scandium leads, served as both electron reservoirs and heat sinks. *C* is the scattering region for charge and energy transport. The red, green, gray, and blue balls refer to the Sc, Se, W, and Mo atoms, respectively. The numbers label the different 2D material layers. Fatbands of the scattering region calculated by (**b**) DFT and (**c**) *GW*. The black, red, and blue curves are the bands projected on the Sc layers, the WSe_2_ layers labeled 1 and 6, and the MoSe_2_ layers labeled 2–5, respectively. (**d**) Contour plot of the LDOS calculated by *GW*. The horizontal axis shows the positions of different layers.

We have scanned a series of vdWHs using first-principles calculations and found two promising structures to have the potential for high performance. In particular, we characterize the thermionic energy conversion performance of one of the devices, namely, the Sc-WSe_2_-MoSe_2_-WSe_2_-Sc, as shown in Fig. 1(a). We predict an equivalent figure of merit, ZT of 3 at temperatures above 600 K for the proposed device and a high cooling performance with the cooling efficiency over 30% of the Carnot efficiency above 450 K.

## Results

### Design principles towards high-performance solid-state thermonics based on vdWHs

There are two crucial factors which can lead to good solid-state thermionic devices: low thermal conductance and suitable thermionic barrier height within several *k*_*B*_*T*. The former is satisfied in vdWHs due to their weak bonding. The thermal conductance can be further reduced by selecting 2D materials with heavier atomic masses. Therefore, WSe_2_, with record low cross-plane thermal conductivity^23^, is a good candidate. The second factor, the barrier height, can be tuned by either using different metal electrodes with different work functions or changing the number of WSe_2_ layers to tune the electron affinity due to the quantum confinement effect. Note that, we should avoid using too thin WSe_2_ layers, namely, mono- or bilayer WSe_2_, to avoid the quantum tunneling effect, which would degrade the device performance. On the other hand, too thick WSe_2_ layers are also not preferred due to the requirement of the ballistic transport. Anderson’s rule, which is a simple and approximate method to estimate the band alignment, does not always predict the correct barrier height^10^. The strong Fermi level pinning effect in 2D materials^17^ rules out this simple method. Therefore, we evaluate the band alignment between WSe_2_ and several metals in the metal-WSe_2_-metal configuration from DFT calculations. During the calculations, the in-plane lattice parameters of the metals (<111> surfaces are used.) are adapted to that of the WSe_2_ layer while the cross-plane lattices are relaxed using the van der Waals functional (methods section). Both the n-type and p-type barrier heights, defined as ${E}_{b}^{n}=CBM-{E}_{F}$ and ${E}_{b}^{p}={E}_{F}-VBM$ where CBM and VBM stands for the conduction band minimum and valence band maximum of the WSe_2_ layers, are summarized in Table 1. As can be seen, the barrier heights with all the metals considered in the metal-WSe_2_-metal configurations are large both for electron and hole transport. The work functions of the metals span a range from 4.2 eV of Al to 6.1 eV of Pt. The ionization potential (IP) of WSe_2_ is calculated to be 4.9 eV. Therefore, Anderson’s rule which would predict ohmic contact for Pt, would not be true according to our calculations. Increasing the WSe_2_ layer thickness would reduce the barrier height which is however still too high for 5 layers. Further increase of the WSe_2_ layer thickness could reduce the barrier height. But thick barriers will violate the ballistic transport condition. Another method which disrupts the direct metal-2D material interactions by inserting a hexagonal boron nitride or graphene layer in between, was found to effectively reduce the barrier height^24,25^. We check this method by inserting a single graphene layer between the Au or Pt and WSe_2_ layers and the corresponding configuration is Au/Pt-G-WSe_2_-G-Au/Pt. Another benefit for this configuration is that introducing graphene could lead to clean interfaces while reducing the thermal conduction further due to the phonon interface scattering. The barrier heights for both Au and Pt covered by graphene show significant reduction compared to those of the pure metal. In particular, the p-type barrier height is found to be only 0.04 eV and 0.02 eV for 3 and 5 layers of WSe_2_, respectively, sandwiched within the Pt/G electrodes. The barrier height is on the order of *k*_*B*_*T*, so Pt-G-WSe_2_-G-Pt is a promising p-type candidate for high-performance solid-state thermionic devices.

**Table 1:** PBE barrier heights of the metal-WSe_2_-metal configurations. G denotes graphene which covers the metal surface, so the corresponding configuration is metal-G-WSe_2_-G-metal. All the numbers are in eV.

WSe_2_ layers	barrier type	Al	Ag	Pd	Pt	Au	Au/G	Pt/G
3	p	0.82	0.70	0.59	0.53	0.49	0.28	0.04
	n	0.51	0.31	0.80	0.72	0.53	0.93	1.13
5	p	0.63	0.58	0.51	0.42	0.37	0.22	0.02
	n	0.40	0.30	0.67	0.68	0.53	0.86	1.04

For the n-type device, first we evaluate the configuration of Sc-WSe_2_-Sc, since Sc has a very low work function of 3.5 eV. The calculated n-type barrier height for the 4 layer WSe_2_ is 0.17 eV which is too high for thermionic energy conversion. Inserting a graphene layer between the Sc and WSe_2_ didn’t show any reduction of the barrier height. A usual way to reduce the Schottky barrier height for conventional metal-semiconductor junctions is through doping. Instead of applying ionic substitutional doping, we find the 2D transition metal dichalcogenide (TMD) family have a staggered band alignment^26^, so charge transfer doping could be obtained by stacking two or more of the TMD layers. MoSe_2_ is used for this purpose since the lattice matches that of WSe_2_, which is favored for the calculations due to the small in-plane supercell. In particular, we evaluate the configuration of Sc-WSe_2_-MoSe_2_-WSe_2_-Sc. For 4 layer MoSe_2_, the calculated barrier height is 0.03 eV which is as promising as that of the p-type Pt-G-WSe_2_-G-Pt. The above discussions are based on the DFT results. In what follows, we show the more accurate GW calculations and the thermionic energy conversion performance of one of the two promising configurations, namely, the Sc-WSe_2_-MoSe_2_-WSe_2_-Sc, due to its smaller supercell size.

### Quasiparticle band structure

The DFT and *GW* band structures of the scattering region of the proposed Sc-WSe_2_-MoSe_2_-WSe_2_-Sc device are shown in Fig. 1(b) and (c) respectively. The fatbands of the WSe_2_ layers (red lines) are significantly distorted from the ideal isolated bands^27^, indicating strong hybridization of the wave functions of WSe_2_ and Sc. This is the result of the short distance of 2.0 Å between Sc and the contacting Se. The band structure of the MoSe_2_ layers (blue lines) are well reproduced^27^, resembling the van der Waals bonding nature. The indirect bandgap from Γ to Q, of the central quadlayer MoSe_2_ was calculated to be 0.90 eV and 1.11 eV and the values for the direct bandgap at K are 1.38 eV and 1.65 eV using DFT and *GW* calculations, respectively. Normally, *GW* predicts much larger bandgap than DFT for 2D materials, e.g., around 1 eV, 0.7 eV, and 0.5 eV larger for monolayer, bilayer, and bulk MoS_2_, respectively^27–30^. Such small correction for the present structure, i.e., 0.2–0.3 eV, reminds us of the significant bandgap renormalization due to the substrate screening effect^19,20^. Note that the renormalization is substrate dependent, e.g., the bandgap of MoS_2_ is reduced by almost 1 eV on Au substrate^20^, while the reduction is only 0.13 eV for MoSe_2_ on bilayer graphene^19^. In the proposed device configuration, the dynamic screening effect is more effective and significant^31^, due to both higher screening of the sandwiched structure compared to MoS_2_ on a substrate (single sided) and the presence of metallic electrodes with substantially higher density of electrons compared to the graphene electrodes.

The Fermi level *E*_*F*_ is located near the conduction bands of MoSe_2_, as shown in Fig. 1(b) and (c), which means MoSe_2_ is n-type doped. We find that the *GW* correction on the transport barrier height is valley dependent. For the K valley, the barrier height is changed from 0.20 eV of DFT to 0.33 eV of *GW*. However, for the Q valley, the correction is only 0.03 eV, i.e., from 0.03 eV to 0.06 eV. Since the large energy difference between the K and Q valleys compared to ${k}_{B}T$, the transport property is dominated by the Q valley. Figure 1(d) shows the *GW* LDOS at each layer. The white area with LDOS of zero is located in the central MoSe2 layers. It spans about 1 eV along the vertical axis, which represents the bandgap of MoSe2. The WSe_2_ layers show metallic behavior as a result of significant wave function hybridizations with Sc. The thermionic transport barrier *E*_*b*_ is determined by the middle MoSe_2_ layers.

### Thermionic transport properties

**Figure 2:**
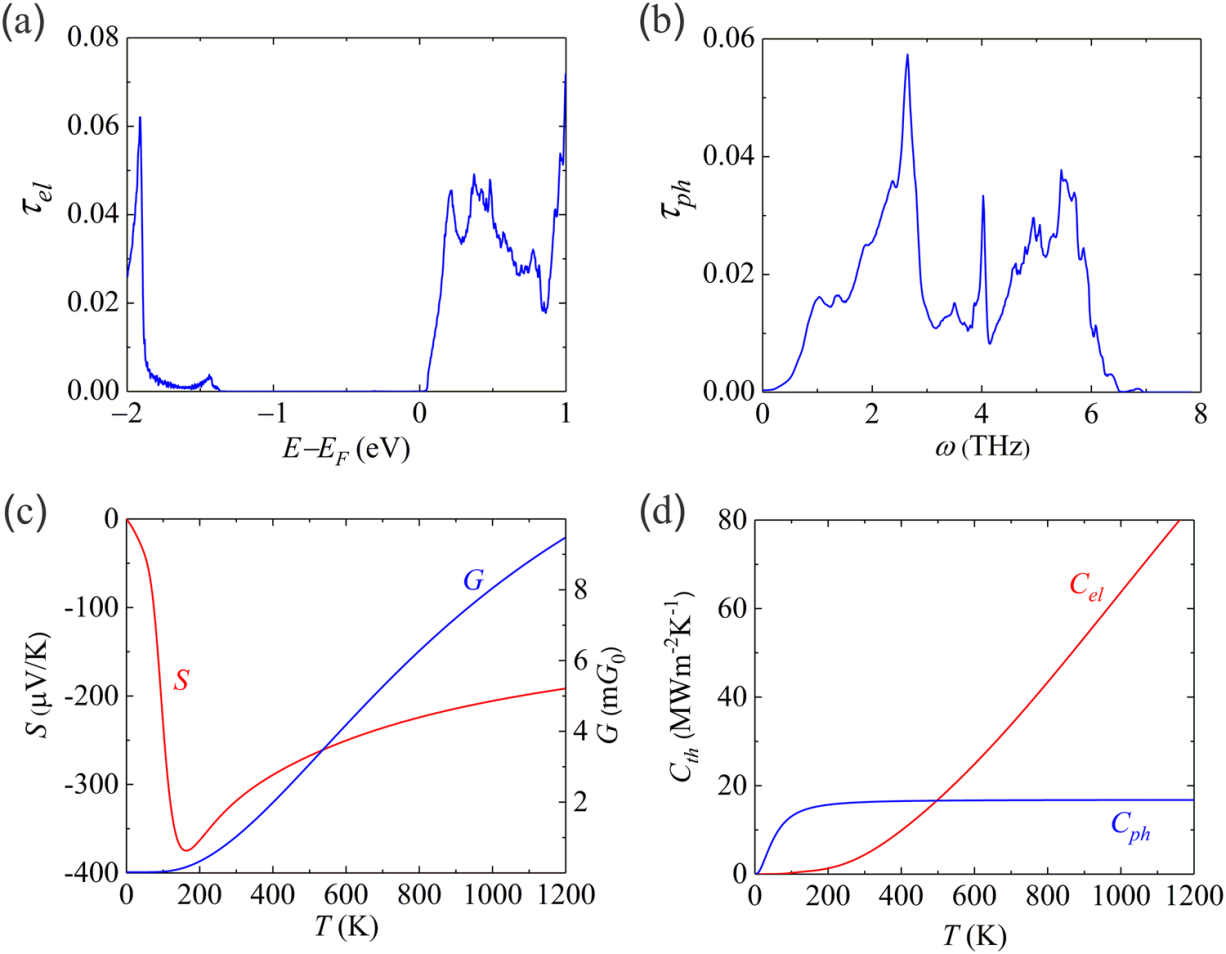
Thermionic transport properties of the proposed 2D vdWH device. (**a**) Electron transmission function *τ*_*el*_, (**b**) phonon transmission function *τ*_*ph*_, (**c**) Seebeck coefficient *S* and electrical conductance *G*, (**d**) thermal conductance *C*_*th*_. *ω* is the phonon frequency and *T* is the absolute temperature. ${G}_{0}=2{e}^{2}/h$ is the electrical conductance quantum.

Figure 2(a) displays the electron transmission function *τ*_*el*_ of the proposed vdWH device. There is a sharp slope of *τ*_*el*_ with respect to energy at 0.06 eV above *E*_*F*_. This slope is due to the Q valley states of the MoSe_2_ layers, consistent with the band structures. In the case of thermoelectric materials, it is known that sharp slopes of the differential conductivity with respect to energy results in asymmetry in electron-hole transport and therefore enhances the Seebeck coefficient. Similarly, sharp slopes of *τ*_*el*_ curve favors the thermionic energy conversion by increasing the asymmetry between low and high energy electrons or in other words by acting as an effective barrier to filter out the low energy electrons. On the hole side, *τ*_*el*_ arises at 1.3 eV below *E*_*F*_. From Fig. 1(c), we know that the valence band maximum (VBM) of the MoSe_2_ layers is located at 1 eV and 1.3 eV below *E*_*F*_ at Γ and K, respectively. Therefore, the holes at Γ do not contribute to the transmission, which can be understood from the small LDOS shown in Fig. 1(d). The *τ*_*el*_ is the electrical conductance *G* at zero temperature. At higher temperatures, electrons have higher kinetic energies and more electrons in Sc can be emitted to the MoSe_2_ conduction bands overcoming the barrier *E*_*b*_, contributing to the increase of *G*, as shown in Fig. 2(c). The n-type doping of the vdWH can be further verified by the negative sign of the Seebeck coefficient *S*. We obtained a maximum *S* of 375 μVK^−1^ at 160 K, while the room temperature value is 320 μVK^−1^.

The phonon transmission function *τ*_*ph*_ is shown in Fig. 2(b). The cutoff frequency ${\omega }_{c}$ of *τ*_*ph*_ is determined by the smallest ${\omega }_{c}$ of the components since three-phonon scattering was not considered. The ${\omega }_{c}$ of the Sc, WSe_2_, and MoSe_2_ layers are 7.5 THz, 8.2 THz, and 10.4 THz, respectively, as determined by the phonon projected DOS (see Figure S1), in agreement with the literature^32–34^. Hence, in the present structure, the highest phonon transport channels are determined by Sc. The phonon thermal conductance *C*_*ph*_ is saturated at 16 MW m^−2^ K^−1^ above 200 K, as shown in Fig. 2(d). This ideal value is four times larger than that of similar configuration of graphene-phosphorene-graphene sandwiched by gold electrodes^10^. This is because gold has lower ${\omega }_{c}$ of 4.7 THz^35^ and there is larger acoustic mismatch in that structure. The electron thermal conductance *C*_*el*_ increases following the trend of *G* as the temperature increased. At room temperature, phonons dominate the thermal transport, until above 500 K, when *C*_*el*_ becomes larger.

**Figure 3:**
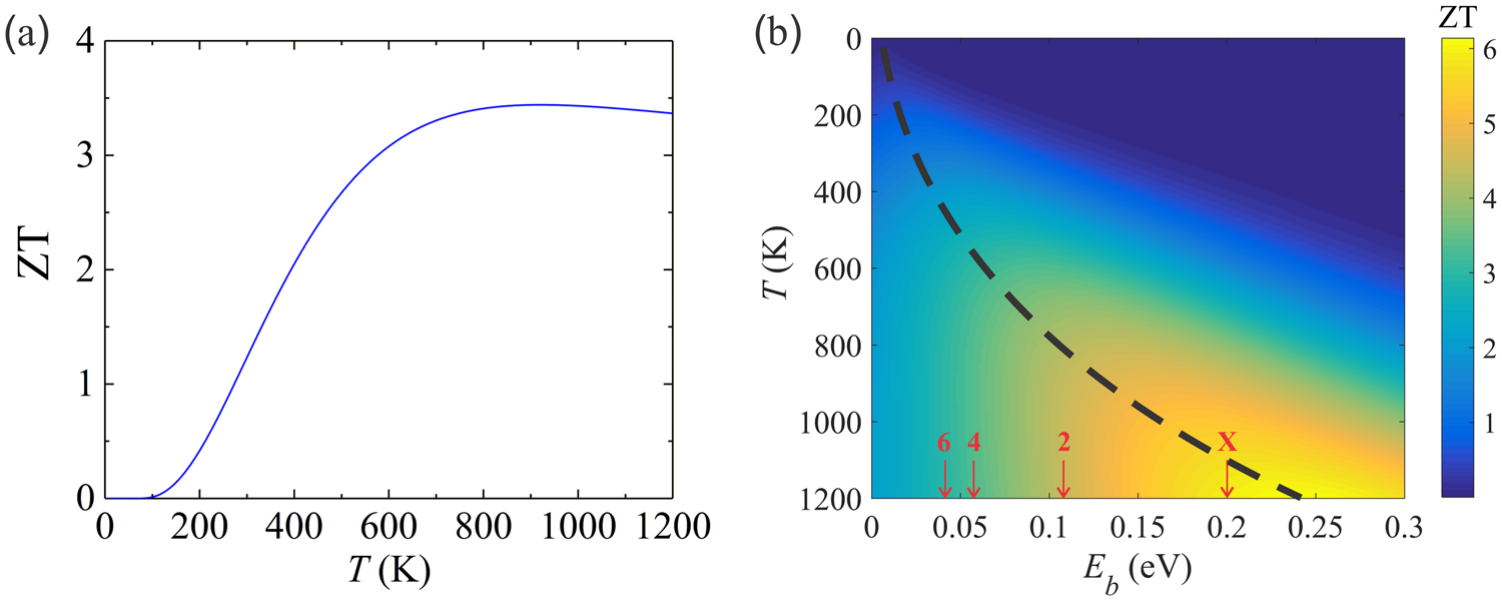
(**a**) The figure of merit ZT of the proposed device. (**b**) Contour plot of ZT as a function of thermionic barrier height *E*_*b*_ and temperature *T*. Energy barrier values labeled by 2, 4, and 6 correspond to different number (n) of MoSe_2_ layers in Sc-WSe_2_-nMoSe_2_-WSe_2_-Sc. Label X is for Sc-4MoSe_2_-Sc. The dashed line shows the position of optimum barrier height leading to maximum efficiency at various temperatures.

We also show the equivalent figure of merit ZT in Fig. 3(a), defined as ${\rm{ZT}}=G{S}^{2}T/({C}_{el}+{C}_{ph})$, to compare with thermoelectric materials. The obtained room temperature ZT is 1.2 which is competitive to the commercial thermoelectric materials with ZT around unity. The ZT increases above 3 at 600 K and reaches a maximum value of 3.4 at 800 K. The experimental record ZT is 2.6 obtained for SnSe at 923 K^36^. Hence, our proposed device is very promising for energy conversion. Since the transport barrier *E*_*b*_ can be tuned by changing the number of MoSe_2_ layers, we plot ZT at varied *E*_*b*_ assuming the same *C*_*ph*_ and shifted *τ*_*el*_, as shown in Fig. 3(b). This approximation was verified by explicitly calculating the transport properties of the WSe_2_–2MoSe_2_-WSe_2_ structure sandwiched by Sc. The *E*_*b*_ of the bilayer MoSe_2_ from *GW* calculation is 0.11 eV, also 0.03 eV larger than that of DFT. The calculated *C*_*ph*_ is 15 MW m^−2^ K^−1^ which is only 1 MW m^−2^ K^−1^ smaller than that of the quadlayer case (see Figure S2). The room temperature ZT is 0.5 which agrees with the plot in Fig. 3(b). For the hexalayer MoSe_2_, the *E*_*b*_ is 0.04 eV after applying a *GW* correction of 0.03 eV (see Figure S3). We label these structures of Sc-WSe2-nMoSe2-WSe2-Sc with the number (n) of the MoSe2 layers in Fig. 3(b). For solid-state thermionic transport, the general constraint on the barrier width $\ell$ is ${\ell }_{t} < \ell  < \lambda$, where $\lambda$ is the electron mean free path and ${\ell }_{t}$ is the minimum thickness to prevent the electron from tunneling through the barrier^1^. For the present structure with 6 layers of MoSe_2_, we have $\ell$ = 4.8 nm, smaller than typical $\lambda$ of 5–10 nm for most semiconductors. For the bilayer MoSe_2_ with $\,\ell$ = 2.4 nm, however, we found electron tunneling (see Figure S4) will degrade the device performance. As shown by the dashed line in Fig. 3(b), the optimum barrier height for the maximum ZT increases as the temperature increased. Label X is for Sc-4MoSe2-Sc which has a *E*_*b*_ of 0.2 eV and a *C*_*ph*_ of 19 MW m^−2^ K^−1^ (see Figures S5 and S2). This structure has a large ZT at very high temperature but nearly zero at room temperature. Note that our calculations are based on ballistic transport regime which may not be valid at very high temperatures.

### Thermionic refrigeration

**Figure 4:**
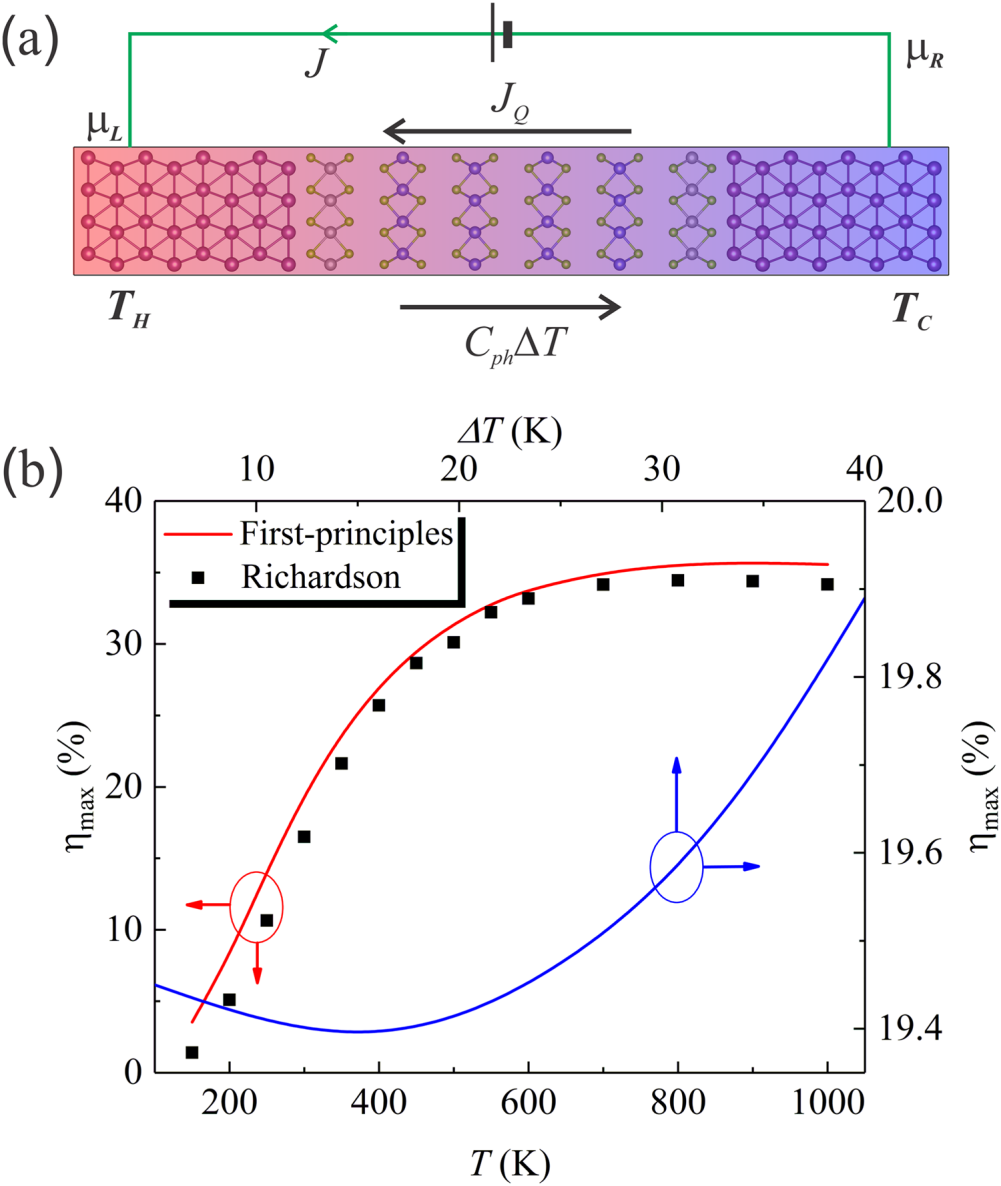
(**a**) Schematic configuration shows the working principle of the proposed device as a thermionic refrigerator. Arrows indicate the charge or energy flow directions. *J*, *J*_*Q*_, *C*_*ph*_*ΔT*, μ, and *T* are electrical current, electron (carriers) thermal current, phonon thermal current, chemical potential, and temperature, respectively. (**b**) Maximum cooling efficiency of the proposed device. The red curve was obtained by fixing the temperature difference ${\rm{\Delta }}T$ to 5 K while varying the hot side temperature *T*_*H*_. For the blue curve, *T*_*H*_ = 300 K was fixed while varying ${\rm{\Delta }}T$. The data calculated based on the Richardson’s law with $\bar{\tau }$ = 1, m^*^ = 1, *E*_*b*_ = 0.06 eV, and *C*_*ph*_ = 16 MW m^−2^ K^−1^, were shown as black squares.

We also propose the present vdWH as a thermionic refrigerator. The working principle is shown in Fig. 4(a). A bias *V* is applied with the chemical potentials ${\mu }_{R} > {\mu }_{L}$ to pump heat *J*_*Q*_ from right to left. As a result of this heat flow a temperature difference ΔT is established between left and right, causing a heat back flow of C_ph_ΔT. The cooling coefficient of performance (COP) is defined as the heat coming out of the cold side divided by the input electrical work: $COP=({J}_{Q}-{C}_{ph}{\rm{\Delta }}T\,)/JV$, where *J* is the electrical current. We have shown that COP can be evaluated from first-principles^10^. We define the cooling efficiency normalized by the Carnot COP for cooling ($CO{P}_{C}={T}_{C}/{\rm{\Delta }}T$) as $\eta =COP/CO{P}_{C}$. The optimum efficiency ${\eta }_{max}$ obtained for the optimum *E*_*b*_ at different working temperatures is shown in Fig. 4(b). ${\eta }_{max}$ follows the same trend as ZT shown in Fig. 3(a). Over 30% of the Carnot efficiency is achieved with the temperature above 450 K. The efficiency changes slightly as ${\rm{\Delta }}T$ increases. At room temperature, ${\eta }_{max}$ increases from 19.45% to 19.89% as ${\rm{\Delta }}T$ increases from 5 K to 40 K.

For thermoelectric materials, the relation between ZT and the efficiency of thermoelectric cooling^1^ is $$(\gamma {T}_{C}-{T}_{H})/$$$$[{\rm{\Delta }}T(\gamma +1)]$$ so that the cooling efficiency with respect to Carnot becomes $${\eta }_{TE}=(\gamma -{T}_{H}/{T}_{C})/(\gamma +1)\approx $$ $$(\gamma -1)/(\gamma +1)$$, where $$\gamma =\sqrt{1+ZT}$$. Take the room temperature ZT of 1.2 of the present structure and $${\rm{\Delta }}T$$ of 1 K, we obtain a $${\eta }_{TE}$$ of 19.3%, almost equal to the thermionic $${\eta }_{TI}$$ of 19.4% obtained from the definition of COP under the same condition. We use this argument to show that the “ballistic” ZT of the thermionic device, which includes the contacts to leads, using the standard definition of the efficiency^1^ leads to almost the same efficiency directly derived from the COP introduced a few lines above. In this sense, the “ballistic” ZT leads to a consistent comparison with thermoelectrics ZT. We also compared our first-principles results with those obtained based on Richardson’s law^1,2^ taking the calculated *E*_*b*_ of 0.06 eV and *C*_*ph*_ of 16 MW m^−2^ K^−1^ as input. We assumed the averaged electron transmission $$\bar{\tau }$$ = 1 and the electron effective mass m^*^ = 1 in the Richardson constant *A*. As can be seen from the Fig. 4(b), the overall agreement is satisfactory. We note that this agreement is fortuitous since the perfect electron transmission is assumed in the Richardson equation. In reality, $$\bar{\tau }$$ should be smaller than 1 due to the back-scattering of the electron waves at the interface and the existence of the barrier; therefore the effective mass must be greater than 1 to reproduce the first-principles results. We note that the device could be further optimized by engineering the phonon thermal conductance, e.g., by introducing lattice mismatch or disorder, since the room temperature thermal conductance is dominated by phonons as shown in Fig. 3(d).

In summary, we have shown how simple design principles and use of accurate first principles calculations, could lead to high-performance for solid-state vdWH thermionic energy conversion devices. we proposed two promising devices, namely, the p-type Pt-G-WSe_2_-G-Pt and n-type Sc-WSe_2_-MoSe_2_-WSe_2_-Sc. The performance of the latter is characterized by more accurate GW and real space Green’ function calculations. We find even though the *GW* correction due to the large dynamic screening effect from the Sc electrodes to the barrier is small, in absolute value (0.03 eV), it still is on the order of the energy barrier itself, and thus strongly affects the thermionic current. The proposed structure has a room temperature ZT of 1.2 which increases to 3 above 600 K. A high performance with cooling efficiency over 30% of the Carnot efficiency above 450 K is predicted. Our findings show that vdWHs with appropriate electrodes have great potential when used in thermionic energy conversion devices.

## Methods

### DFT calculation

The DFT calculations were performed using the Quantum Espresso package^37^. SG15 norm-conserving pseudopotentials^38^, PBE^39^ exchange correlation functional, kinetic energy cutoff of 60 Ry, and a k mesh of 12 × 12 × 1 were used. Lattice was relaxed with the force on each atom less than 1.0e-4 Ry/Bohr. We first optimized separately the lattices of Sc, WSe_2_, and MoSe_2_. The optimized in-plane lattice constants are 3.319 Å, 3.320 Å, and 3.320 Å, respectively. We then constructed the vdWH including the electrodes with AB stacking by fixing the in-plane lattice constant of 3.319 Å across all the layers. In this configuration, there are almost no in-plane strains on any layer. With the in-plane lattice constant fixed, we further optimized the whole structure using optB86 functional^40^ to include the vdW correlations. The optimized interlayer distances of MoSe_2_-MoSe_2_ and MoSe_2_-WSe_2_ are 6.5 Å. The relaxed coordinates can be found in supplementary files. DFT band structure calculations were performed on the relaxed structure but with PBE functional. Spin-orbit coupling (SOC) was not included since the effect is less important for Mo than W, given that the thermionic barrier is determined by the MoSe_2_ layer. Moreover, SOC is less important for CBM than for VBM. Even for the CBM, the Q valley is less affected than the K valley (see Figure S6). Fatbands were calculated using the maximally localized Wannier functions (MLWFs) as implemented in the wannier90 code^41^. Initial projections for Sc, Se, W, and Mo atoms were chosen as (*d*; *sp*^3^−1), *p*, *d*, *d*, respectively.

### *GW* calculation

Single-shot *GW* or *G*_0_*W*_0_ calculations based on PBE wave functions were performed using the ABINIT code^42,43^. SG15 norm-conserving pseudopotentials, wave function cutoff of 60 Ry, dielectric matrix cutoff ${{\epsilon }}_{C}$ of 10 Ry, exchange self-energy cutoff of 240 Ry, 1500 bands for both the dielectric matrix and self-energy calculations, and a k mesh of 9 × 9 × 1 were used. We included 4 layers of Sc at each side (total 8 layers) in the *GW* calculation. The transport barrier did not change with 8 more Sc layers added. The contour deformation method with 10 grids on the imaginary axis, 20 grids on the real axis, and the cutoff frequency of 1 Ry was used to calculate the dielectric matrix and self-energy. Further increasing the imaginary axis grid to 20 and real axis grid to 50 changed the results by less than 1%. *GW* calculations highly depend on the convergence of the parameters, of which the most important are k mesh, ${{\epsilon }}_{C}$, and the number of bands (nbands). The latter two are interdependent. We checked the convergence of k mesh with ${{\epsilon }}_{C}$ = 10 Ry, nbands = 500, and plasmon-pole model used. Increasing the k mesh from 9 × 9 × 1 to 12 × 12 × 1 changed the barrier height by only 3 meV. The convergence of ${{\epsilon }}_{C}$ and nbands were checked with k meshes of 3 × 3 × 1 and 6 × 6 × 1 (see Figure S7). The accuracy with error within 0.01 eV can be obtained with ${{\epsilon }}_{C}$ = 20 Ry and nbands = 3000. However, using these values for the 9 × 9 × 1 k mesh is computationally prohibitive. We found that the convergence curves at different k meshes with the same ${{\epsilon }}_{C}$ are quite similar. Therefore, we first evaluated the barriers with ${{\epsilon }}_{C}$ = 10 Ry, nbands = 1500, and ${{\epsilon }}_{C}$ = 20 Ry, nbands = 3000 for k mesh of 6 × 6 × 1. The corresponding barrier heights are *b*_1_ and *b*_2_. We then calculated the barrier height *b*_3_ with ${{\epsilon }}_{C}$ = 10 Ry and nbands = 1500 for k mesh of 9 × 9 × 1. The final correct barrier height was estimated using *b*_3_ + *b*_2_ – *b*_1_. For our present calculations, we found that the correction *b*_2_ – *b*_1_ was very small, i.e., −0.03 eV. Thus we used the result from ${{\epsilon }}_{C}$ = 10 Ry and nbands = 1500 to plot the band structure, but the corrected bands were used in the transport calculations. The *GW* band structure was generated using the MLWFs.

### Electron transport

We used the real space Green’s function method^44,45^ with localized basis Hamiltonian constructed using MLWFs to calculate the ballistic electron transport. The retarded Green’s function of the scattering region reads:

1 $${{\boldsymbol{G}}}^{r}={[(\varepsilon +i\delta ){\boldsymbol{I}}-{\boldsymbol{H}}-{{\boldsymbol{\Sigma }}}_{L}-{{\boldsymbol{\Sigma }}}_{R}]}^{-1}$$

where ***I*** the identity matrix, $\varepsilon$ the electron energy, *iδ* is a small imaginary number. ${{\boldsymbol{\Sigma }}}_{L,R}$ denotes the self-energy of the left (*L*) or right (*R*) lead. ***H*** is the scattering region Hamiltonian, on which we imposed a minor scissor correction of 0.03 eV to take the *GW* correction into account. The electron transmission function is ${\tau }_{el}={\rm{Tr}}({{\boldsymbol{G}}}^{r}{{\boldsymbol{\Gamma }}}_{L}{{\boldsymbol{G}}}^{a}{{\boldsymbol{\Gamma }}}_{R})$, where ${{\boldsymbol{G}}}^{a}={({{\boldsymbol{G}}}^{r})}^{\dagger }$ is the advanced Green’s function and ${{\boldsymbol{\Gamma }}}_{L/R}=i({{\boldsymbol{\Sigma }}}_{L/R}-{{\boldsymbol{\Sigma }}}_{L/R}^{\dagger })$. Note that the above method is used for 1D transport. For the case of 3D transport, one samples the 2D Brillouin zone perpendicular to the transport direction with a fine k mesh. For each transverse k point, the k-dependent transmission ${\tau }_{el}(\varepsilon ,k)$ should be calculated and the total transmission would be ${\tau }_{el}(\varepsilon )=\sum _{k}{\tau }_{el}(\varepsilon ,\,k){w}_{k}\,$, where *w*_*k*_ is the k point weight. In the present calculation, we used a k grid of 150 × 150 × 1 to obtain a smooth transmission function. The electron transmission was calculated using the WanT code^46^. With ${\tau }_{el}$, one can obtain the coherent transport coefficients under the linear response approximation^47^:

2 $$G={e}^{2}{L}_{0}$$

3 $$S={L}_{1}/(eT{L}_{0})$$

4 $${\kappa }_{el}=({L}_{2}-{L}_{1}^{2}/{L}_{0})/T$$

5 $${L}_{n}=2/h{\int }^{}d\varepsilon {\tau }_{el}(\varepsilon )\times {(\varepsilon -\mu )}^{n}\times (\,-\,\partial f/\partial \varepsilon )$$

where *f* is the Fermi-Dirac distribution function. The two-probe electrical current and electron thermal current are:

6 $$J=2e/h{\int }^{}d\varepsilon {\tau }_{el}(\varepsilon )({f}_{L}-{f}_{R})$$

7 $${J}_{Q}=2/h{\int }^{}d\varepsilon {\tau }_{el}(\varepsilon )(\varepsilon -\mu )({f}_{L}-{f}_{R})$$

We can impose any two of the four quantities ($J,{J}_{Q},{\rm{\Delta }}T,\,{\rm{\Delta }}\mu$) and obtain the remaining two from the above two equations.

### Phonon transport

The phonon Green’s function method^48,49^ was used to calculate the ballistic phonon transport. The equations to calculate the phonon transmission function ${\tau }_{ph}$ are similar to those of the electron transport. One only needs to substitute $\varepsilon$ by *ω*^2^ and ***H*** by ***Φ***, where ***Φ*** is the interatomic force constant matrix divided by atomic masses. The phonon thermal conductance was calculated as:

8 $${C}_{ph}=\hslash /2\pi {\int }^{}d\omega \omega {\tau }_{ph}(\omega )(\partial n/\partial T)$$

where *n* is the Bose-Einstein distribution function. We used the finite difference method as implemented in the SIESTA code^50^ to calculate the interatomic force constant matrix. A 3 × 3 × 1 supercell was constructed and the displacement was 0.01 Å for each degree of freedom. Troullier-Martins pseudopotentials^51^, double zeta plus polarization basis set, PBE exchange correlation functional, a 6 × 6 × 1 k mesh, energy shift of 50 meV, and real space grid cutoff of 300 Ry were used for the supercell calculations. With the calculated interatomic force constant matrix as input, the phonon transmission was calculated using the transport module of the WanT code. The phonon PDOS was evaluated using the Phonopy code^52^.

## Electronic supplementary material

Supplementary information
