# Supplementary material for: High-Performance Solid-State Thermionic Energy Conversion Based on 2D van der Waals Heterostructures: A First-Principles Study
Source: Sci Rep. 2018 Jun 18;8:9303. doi: 10.1038/s41598-018-27430-0 (PMC6006252; doi:10.1038/s41598-018-27430-0)
Supplement: Supplementary file 1 — Supplementary information [file 41598_2018_27430_MOESM1_ESM.pdf]

Supplementary for

High-Performance Solid-State Thermionic Energy Conversion Based on 2D van der Waals Heterostructures: A First-Principles Study

Xiaoming Wang[1,2], *Mona Zebarjadi*[3,4], *Keivan Esfarjani*[4,5,6*]

[1]Department of Physics and Astronomy, and [2]Wright Center for Photovoltaic Innovation and Commercialization, The University of Toledo, Toledo, Ohio 43606, United States

[3]Department of Electrical and Computer Engineering, [4]Department of Materials Science, [5]Department of Mechanical and Aerospace Engineering, and [6]Department of Physics, University of Virginia, Charlottesville, Virginia 22904, United States

*Email: k1@virginia.edu

Effects of the graphene or WSe_2 thickness of the transport

In our prosed structure, namely, Pt-G-WSe2-G-Pt and Sc-WSe2-MoSe2-WSe2-Sc, single layer of graphene or WSe_2 is inserted between the metal electrode and semiconductors. However, for experiments, few-layer graphene or WSe_2 is usually inserted due to the challenges in transfer of sample. We note here that although the graphene or WSe_2 could reduce the thermal conductance due to phonon interface scattering, inserting more layers of graphene or WSe_2 would not reduce the thermal conductance further significantly. Since we are dealing with ballistic transport, in which regime the thermal conductance is independent on the layer thickness. Indeed, from Fig. S2 of the supporting information, we can see that the thermal conductance change is quite small for different thickness of $MoSe_2$.

On the contrary, inserting more layers of graphene or WSe_2 could change the electron transport. For electron transport which is in the ballistic regime, the barrier height is the most important parameter. The work function of Pt/G is 4.86 eV [Adv. Electron. Mater. 2016, 1500405], while the work function of graphite is 4.62 eV [Nature 169, 702–703 (1952)]. Thus, inserting more layers of graphene, the work function tends to decrease, resulting in larger p-type barrier height which is unfavorable for thermionic energy conversion. For WSe_2 layer, it hybridizes strongly with Sc, as shown in Fig. 1 of the manuscript, resulting in metallic behavior of the first layer. If inserting one more WSe_2 layer, then this second layer would have no hybridization and show a bandgap. Since the electron affinity of WSe_2 is smaller than $MoSe_2$, it will produce a larger n-type Schottky barrier. If only one more WSe_2 layer is inserted, electrons can still tunnel through it, but with a degraded electron transmission function. For more WSe_2 layers, the overall barrier height would be larger, which would prohibit electron transport.

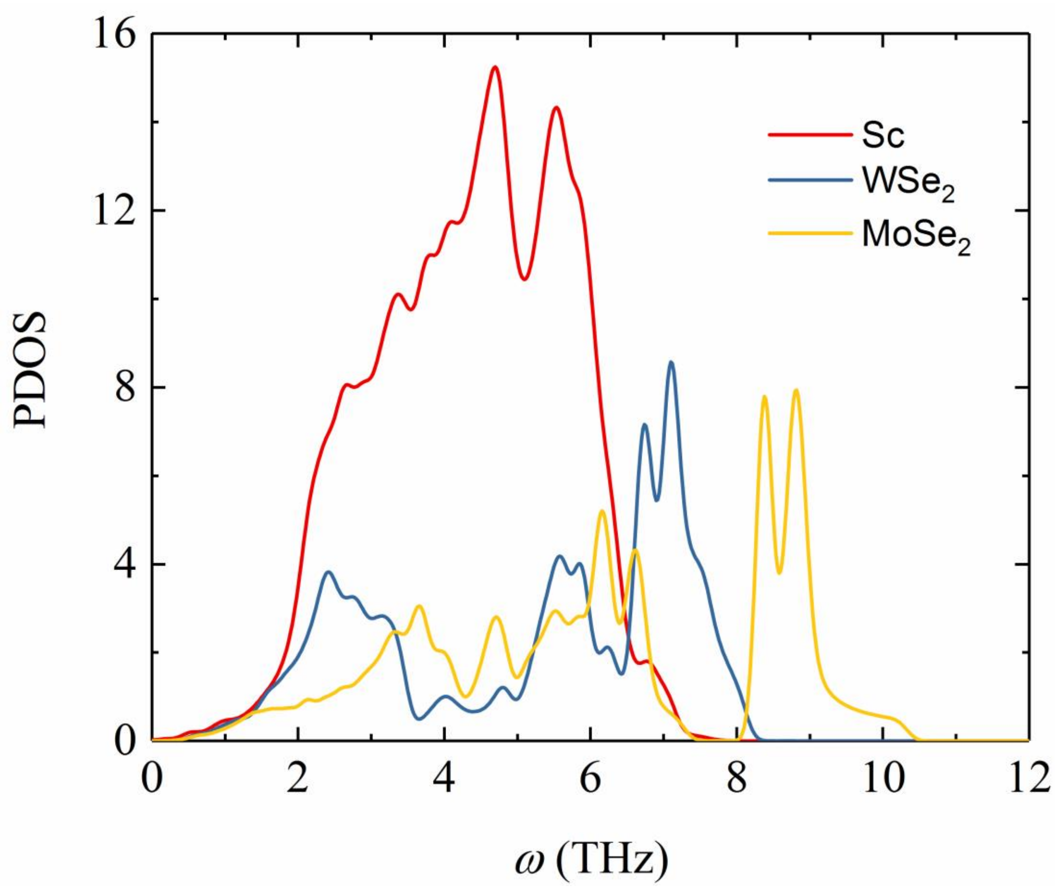

Figure S1. Phonon PDOS of the Sc-WSe_2-4$MoSe_2$-WSe_2-Sc heterostructure.

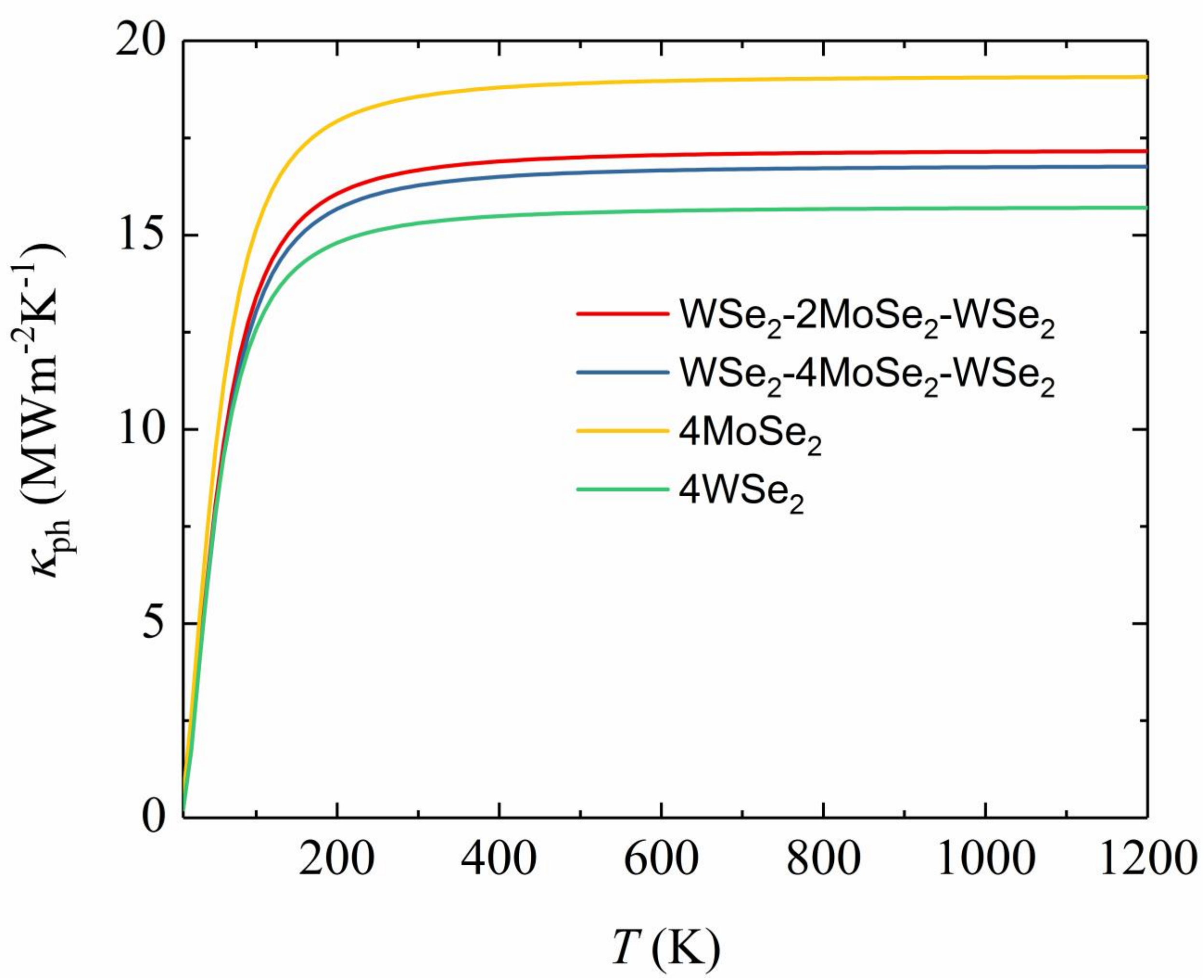

Figure S2. Phonon thermal conductance of different structures sandwiched by Sc electrodes.

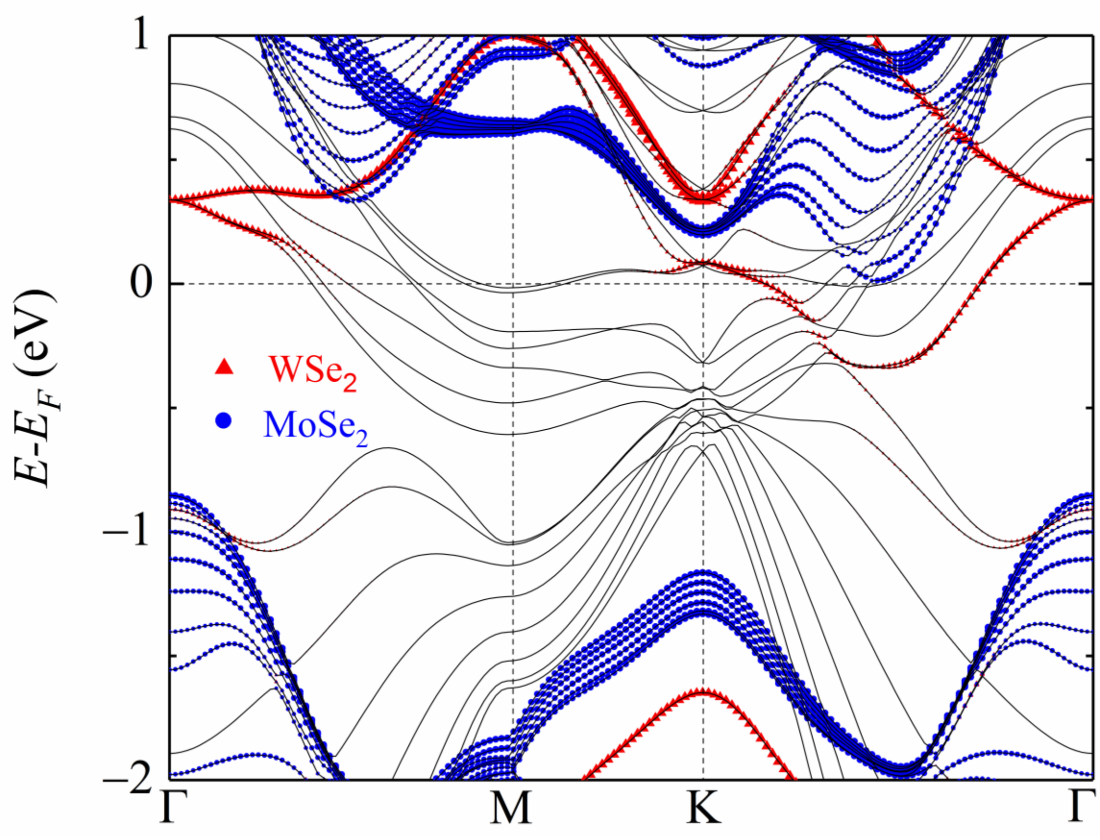

Figure S3. DFT fatbands of Sc-WSe_2-6$MoSe_2$-WSe_2-Sc. The DFT barrier is 0.01 eV.

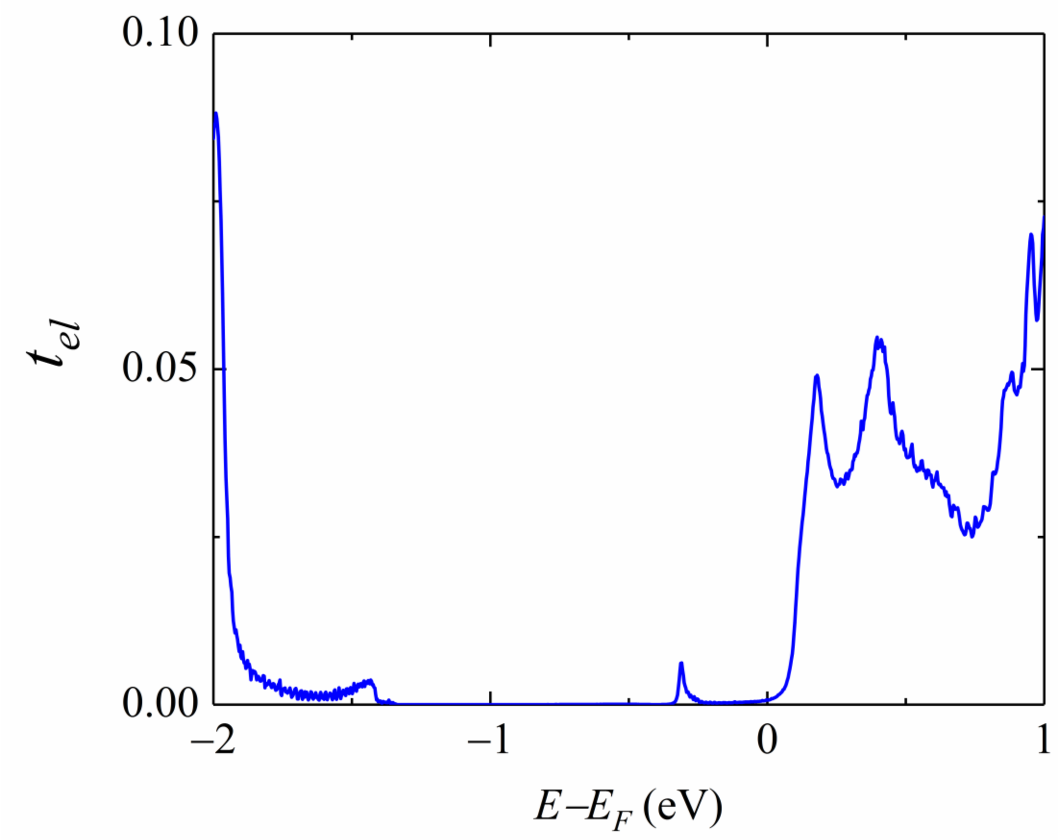

Figure S4. Electron transmission function of Sc-WSe_2-2$MoSe_2$-WSe_2-Sc. The peak at -0.3 eV and the nonzero transmission at Fermi level indicates the quantum tunneling effect.

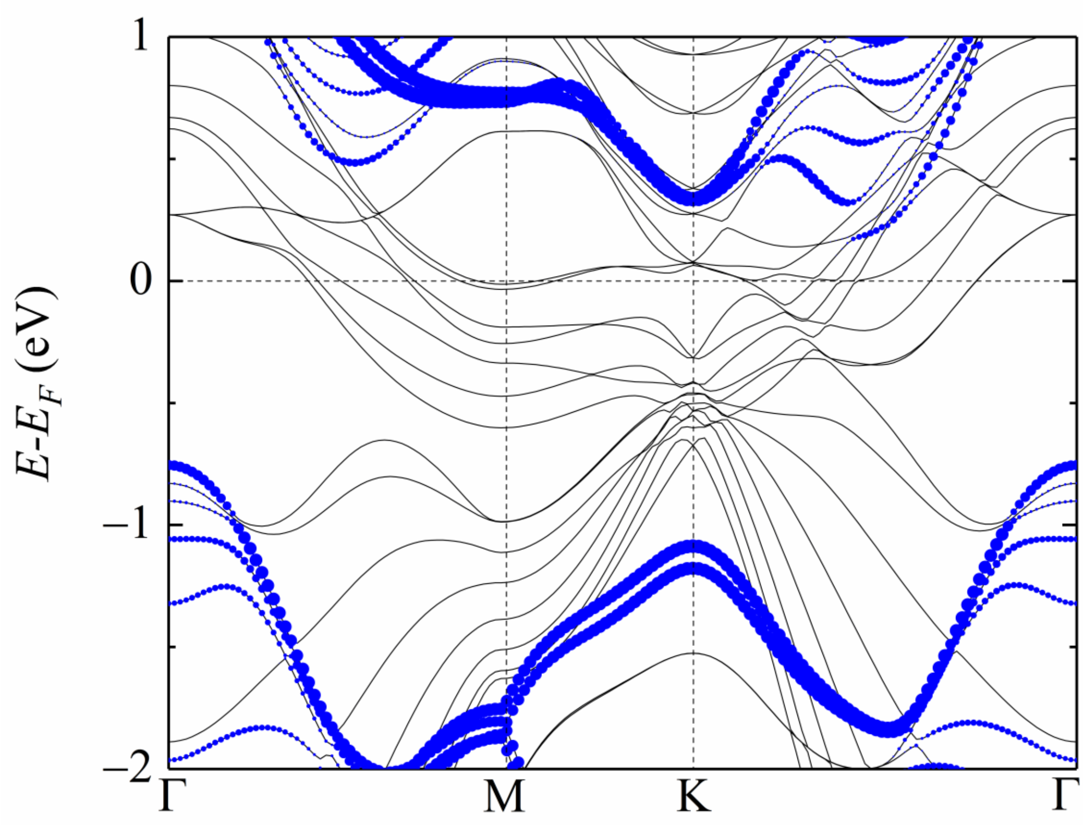

Figure S5. DFT band structure of Sc-4MoSe$_2$-Sc. The bands of MoSe2 layers are shown by blue dots. This configuration has a DFT barrier height of 0.17 eV.

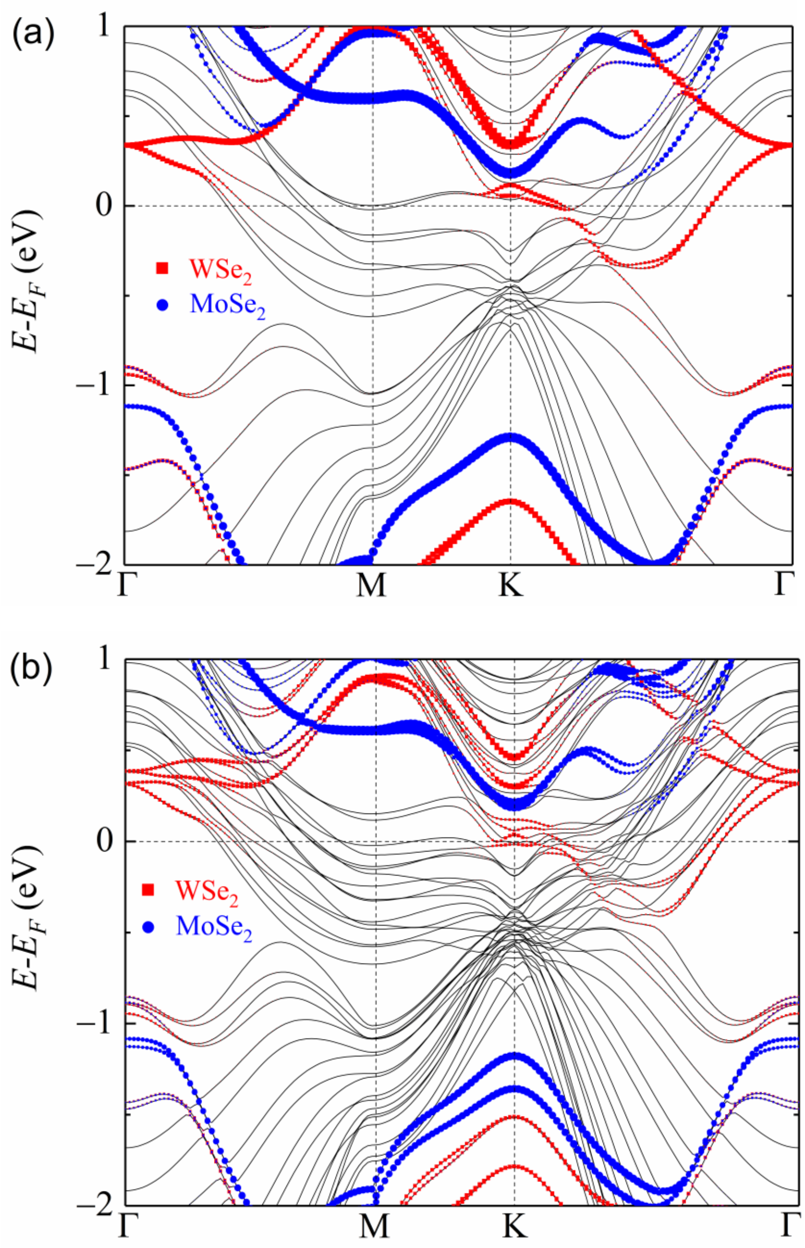

Figure S6. DFT fatbands of Sc-WSe_2-$MoSe_2$-WSe_2-Sc (a) without and (b) with spin-orbit coupling effect. SOC mainly affects the VBM at K. For the Q and K valleys at CBM, the energy change is less than 0.01 eV.

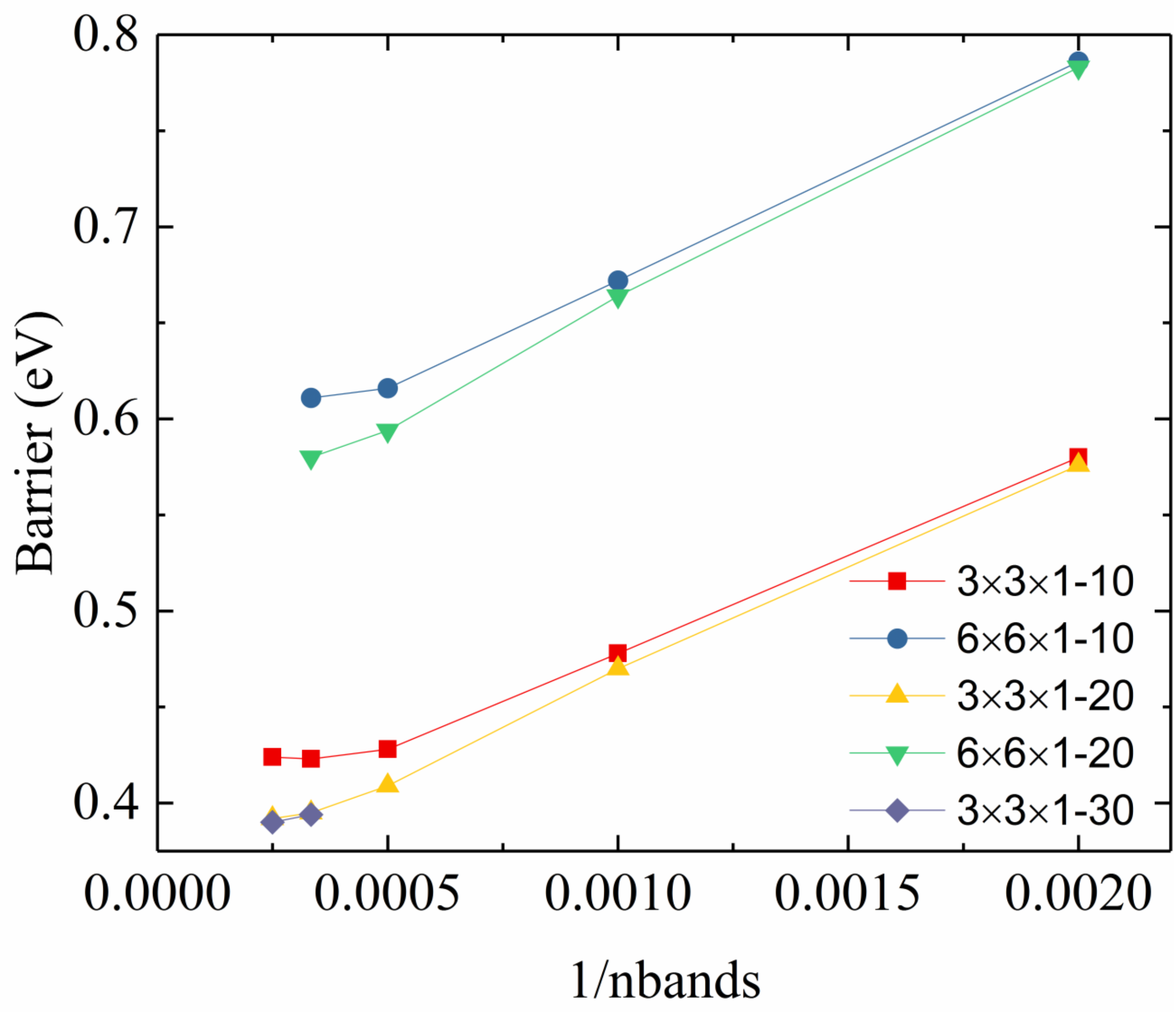

Figure S7. Convergence study on the nbands and ϵ_C (in Ry) for the *GW* calculations with different k meshes. The legend format is [k mesh - ϵ_C].
